# Fetoscopic Myelomeningocele (MMC) Repair: Evolution of the Technique and a Call for Standardization

**DOI:** 10.3390/jcm14051402

**Published:** 2025-02-20

**Authors:** Stephanie M. Cruz, Sophia Hameedi, Lourenco Sbragia, Oluseyi Ogunleye, Karen Diefenbach, Albert M. Isaacs, Adolfo Etchegaray, Oluyinka O. Olutoye

**Affiliations:** 1 The Fetal Center, Nationwide Children’s Hospital, Columbus, OH 43205, USA; stephanie.cruz@nationwidechildrens.org (S.M.C.); oluseyi.ogunleye@nationwidechildrens.org (O.O.); karen.diefenbach@nationwidechildrens.org (K.D.); albert.isaacs@nationwidechildrens.org (A.M.I.); adolfo.etchegaray@nationwidechildrens.org (A.E.); 2 Department of Pediatric Surgery, Nationwide Children’s Hospital, Columbus, OH 43205, USA; sophia.hameedi@nationwidechildrens.org; 3 Center for Regenerative Medicine, Abigail Wexner Research Institute, Nationwide Children’s Hospital, Columbus, OH 43205, USA; lourenco.neto@nationwidechildrens.org; 4 Division of Pediatric Surgery, Ribeirão Preto Medical School, University of São Paulo, Sao Paulo 14048-900, Brazil; 5 Section of Maternal Fetal Medicine, Department of Obstetrics and Gynecology, Nationwide Children’s Hospital, Ohio State University College of Medicine, Columbus, OH 43210, USA; 6 Department of Surgery, The Ohio State University College of Medicine, Columbus, OH 43210, USA; 7 Department of Neurosurgery, Nationwide Children’s Hospital, Columbus, OH 43205, USA

**Keywords:** myelomeningocele, fetal repair, fetoscopy, spina bifida, MMC

## Abstract

Fetal surgery has made significant strides over the past 40 years, facilitated by advances in technology and imaging modalities enabling the diagnosis and treatment of congenital anomalies in utero. The Management of Myelomeningocele Study (MOMS), a multicenter randomized controlled trial, established open fetal myelomeningocele (MMC) repair as the gold standard for improving neurological outcomes compared to postnatal repair. However, this approach is associated with increased maternal complications and preterm birth due to hysterotomy, prompting the exploration of minimally invasive alternatives. Due to the lack of an existing randomized control trial with fetoscopic MMC repair and variations in technique (percutaneous versus laparotomy/transuterine access, different trocar configurations, closure methods, and patch applications) among different fetal centers, more studies are needed to optimize this approach as an alternative to the standard of care. This paper proposes to assess the basics tenets of open fetal MMC repair and to establish guiding principles for a fetoscopic approach that could prove to be equivalent or superior to open fetal MMC repair in maternal and fetal outcomes and lead to clinical implementation.

## 1. Introduction

### 1.1. Background

Myelomeningocele (MMC) is a common birth defect affecting 1 in 1000 live births worldwide [1,2]. Approximately 31% to 97% of pregnancies, depending on the country, are electively terminated following prenatal diagnosis of MMC [3]. Although survival rates have improved in the modern era, ranging from 60% to 90% in developed countries and about 50% in underdeveloped regions, there continues to be significant association with morbidity [4,5].

Though the exact etiology of MMC remains to be understood, it is hypothesized that the failure of neural tube or mesenchymal closure during embryogenesis results in the characteristic protrusion of meninges and spinal cord through open vertebral arches. This leaves the neural tissue unprotected and vulnerable to damage from exposure to amniotic fluid and physical trauma in utero [1]. Hydrocephalus and hindbrain herniation result in significant morbidity as well as motor and sensory deficits from spinal cord maldevelopment and injury. The majority of patients with MMC have an Arnold-Chiari II malformation, which consists of hindbrain herniation through the foramen magnum secondary to cerebrospinal fluid (CSF) leakage through the spinal cord defect.

### 1.2. Rationale for Fetal Intervention

Historically, treatment for MMC primarily focused on surgical closure at birth and supportive care, both of which were associated with significant long-term morbidity. The existence of neurological sequelae despite prompt postnatal surgical intervention suggests that there is significant damage to the spinal cord in the prenatal period. The “two-hit” hypothesis suggests that neural damage in MMC occurs in two stages. The first hit involves abnormal spinal cord development during early embryogenesis, while the second hit results from continuous in utero exposure of the spinal cord to physical trauma and chemical damage from the amniotic fluid [6].

There are significant clinical observations that support this theory. Post-mortem pathologic analyses of fetuses with MMC have demonstrated damage and loss of neural tissue at the site of the lesion, with normal tissue proximally suggesting that tissue injury may occur after primary neural tube formation [7]. Multiple studies have examined fetal leg movement as a marker for spinal cord function and, when comparing prenatal versus postnatal function, found a significant increase in abnormal leg movements postnatally, which may indicate progressive spinal cord damage through the course of gestation [8,9]. Lastly, studies have shown that infants with MMC who undergo cesarean delivery prior to the onset of labor and rupture of membranes have improved motor function at 2 years of age, suggesting that there may be some aspect of traumatic injury to the exposed neural tissue from the loss of amniotic fluid during labor [6,10,11].

Animal models of MMC have been developed in various species to assess whether fetal intervention can reduce progressive spinal cord damage and associated long-term neurologic morbidity. Initial models using primates, rodents, and piglets have been focused on the immediate repair of surgically created or chemically induced spina bifida, and they have demonstrated improved neurologic function following in utero spinal cord coverage; however, the generalizability of these findings to clinical practice has been limited since there was no time interval between defect creation and closure [12,13,14]. A sheep model of surgically created MMC was developed in the 1990s that more closely recapitulated human disease on a clinical and histopathological level. The spina bifida lesion was created at 75 days of gestation and then repaired at 100 days of gestation using a reversed latissimus dorsi flap to cover the exposed placode. The sheep were then delivered via cesarean section at 140 days of gestation (term: 145 days) and were assessed for neurologic function and histologic abnormality. The fetal repair group demonstrated near normal neurologic function and histologic architecture compared to the unrepaired group, which suffered from paraplegia, incontinence, and loss of sensation, with significant neural tissue loss seen on histology [6,15,16,17]. Further studies on the sheep model have also shown that in utero repair reverses hindbrain herniation and its deleterious downstream effects [18].

These observations from clinical and experimental studies support the utility of in utero intervention in MMC. The goals of surgical correction in the prenatal period are to reduce damage to spinal cord structures from exposure to amniotic fluid, reverse hindbrain herniation, and reduce hydrocephalus, thereby minimizing the need for shunting, reducing loss of function due to tethering, and improving motor function and quality of life for children with MMC. This review aims to assess the basic principles of open fetal MMC repair and to establish guidance for a fetoscopic approach with the goal of promoting standardized clinical implementation that could prove to be equivalent or superior to open fetal MMC repair in maternal and fetal outcomes.

## 2. Open Fetal MMC Repair

At the advent of fetal surgery in the late 1990s, only patients with life-threatening defects and poor predicted outcomes were considered candidates for fetal intervention. With the development of advanced imaging modalities, refinements in surgical technique and improvement in maternal morbidity, in utero intervention became accessible for those with non-lethal but severe disease. Through experience with fetal intervention and insights gleaned from animal models, it was established that repair between 19 and 25 weeks was optimal to reduce the duration of spinal cord exposure to amniotic fluid while balancing the feasibility of manipulating delicate fetal tissue [6].

In utero MMC closure is technically more difficult than traditional postnatal closure. Special considerations for fetal MMC closure include a smaller operative field, which is limited by a narrow uterine window; smaller and more delicate structures; the careful handling of the placode, which is still functional in the prenatal period; minimizing blood loss to avoid the need for fetal transfusion; and minimizing operative time to limit fetal exposure to the extrauterine environment [19]. Further, fetal surgery requires a coordinated team of specialists from various fields, including neurosurgery, pediatric/fetal surgery, maternal–fetal medicine, anesthesiology, and fetal cardiology, and obstetric and pediatric nursing staff who must all work together to ensure the safety and efficacy of the procedure for both the mother and the fetus.

The open in utero repair procedure begins with maternal laparotomy followed by a hysterotomy, typically performed using a uterine stapling or appropriate sealing device. Once the uterus is opened, the fetus is carefully positioned so that the MMC defect is centered within the uterine incision. It is critical to stabilize the fetus with minimal pressure to avoid fetal cardiac dysfunction. The umbilical cord is positioned at a safe distance away from the defect. A soft rubber catheter connected to a rapid infusion device infuses warm lactated Ringer’s solution into the uterus to maintain fetal warmth and buoyancy. A fetal anesthetic cocktail consisting of a narcotic, an anticholinergic, and a muscle relaxant is administered intramuscularly to provide additional anesthesia, analgesia, and muscular relaxation and to prevent bradycardia due to surgical stimulation [19].

Once the fetus is appropriately positioned and anesthetized, the MMC defect is closed. Various techniques have been utilized to close the fetal myelomeningocele defect. The initial approach of merely closing the skin over the placode to provide a protective barrier is no longer thought to be sufficient. A technique shown to have excellent outcomes is the three-layer closure (3LC) [20]. First, the placode is dissected off the adjacent sac and epithelial tissue, which is excised up to the skin edges. The skin is excised from the placode circumferentially by cutting into the arachnoid layer and releasing the connections to the MMC sac. Once the placode and spinal cord are released from the sac, the placode is inspected, and any residual epidermal tissue is trimmed away to minimize the risk of epidermoid/dermoid inclusion cyst formation. The manipulation of the fragile fetal placode is minimized as much as possible to avoid traumatic injury to functional nervous tissue, therefore re-neurulation is not attempted. A multiple-layer closure is performed similar to postnatal repair. The dura is dissected free and closed over the placode using a running 4-0 PDS suture. A dural patch may be used to strengthen this closure based on surgeon discretion and preference. Next, the myofascial layer is exposed via extensive undermining of the skin from the defect site, which will allow for skin closure with minimal tension. Lateral elliptical incisions are made bilaterally in the myofascial layer, which is then undermined and rotated to create myofascial flaps; the flaps are then closed over the dural closure using a running 4-0 PDS stitch. Lastly, the surrounding skin is trimmed to healthy tissue and delicately approximated to close the defect; the skin is closed in a single layer using a running 4-0 PDS suture. Achieving tension-free skin closure can be challenging, particularly for defects greater than 3 cm in maximal width. Compared to postnatal repair, in utero skin closure requires a larger full-thickness stitch to prevent tearing of the thin fetal skin, especially in fetuses at less than 23 weeks of gestation. For larger defects that cannot be closed primarily without tension, an acellular dermal graft can be used to bridge the skin or lateral relaxing incisions can be made on the skin of the flank to permit the mobilization and closure of the defect. Rotation flaps can also be created to close a large skin defect. Once the fetal defect is repaired, the uterus is refilled with lactated Ringer’s solution to an appropriate volume, as measured by the deepest vertical pocket on ultrasound. Antibiotics are administered into the amniotic cavity, and the maternal hysterotomy and laparotomy sites are closed [19].

### MOMS Trial

In light of the promising results of fetal intervention for MMC in animal models and the initial case series, the Management of Myelomeningocele Study (MOMS), a pivotal multicenter randomized clinical trial, was designed with the crucial objective of comparing outcomes of prenatal and postnatal surgical repair of MMC [21]. The trial was conducted at three major maternal–fetal surgery centers—The Children’s Hospital of Philadelphia, Vanderbilt University, and University of California, San Francisco—with a fourth center (George Washington University) as the independent data review center. This study aimed to enroll a total of 200 eligible women over the seven-year study period, from February 2003 to December 2010.

Primary outcomes were assessed at 12 and 30 months of age. All children underwent evaluations of physical characteristics, neurological examinations, and developmental tests at these intervals. The first primary outcome, assessed at 12 months, was a composite of fetal or neonatal death or the need for a cerebrospinal fluid (CSF) shunt. The second primary outcome, assessed at 30 months, was a composite score of the Mental Development Index of the Bayley Scales of Infant Development II and the child’s motor function, adjusted for the level of injury.

Secondary outcomes included maternal surgical and obstetric complications, as well as neonatal morbidity and mortality. In infancy, secondary outcomes focused on the radiographic appearance of Chiari II malformation, time to first shunt placement, time to achieve independent locomotion, and the Motor and Psychomotor Development Index.

A standardized surgical technique was employed at all three centers, though it has since evolved following the completion of the study. Initially, the placode was carefully dissected from the surrounding tissue and dropped into the spinal canal, and then, the dura mater was closed, and finally, the skin. If it was not possible to close the dura, a biologic dural graft (Duragen; Integra Life Sciences Corporation, Plainsboro, NJ, USA) was used as a substitute. Finally, the skin was closed with a thin running monofilament suture. If the skin could not be approximated without tension, releasing incisions or an acellular dermal matrix (Alloderm; Life Cell, Branchburg, NJ, USA) was utilized.

The trial was concluded early after the recruitment of 183 out of the planned 200 patients, as an interim analysis demonstrated the efficacy of prenatal surgery. The results were reported based on 158 patients evaluated at 12 months and 134 patients with 30-month follow-up. The baseline demographic data were similar between fetal and neonatal correction, though the fetal surgery group had a lower proportion of female fetuses and a lower lesion level on ultrasonography (*p* = 0.02).

In terms of maternal complications, there were no reported maternal deaths. However, the group that underwent fetal surgery had higher rates of spontaneous membrane rupture, chorioamniotic separation, oligohydramnios, and placental abruption. At the time of delivery, one-third of patients had an area of dehiscence or a very thin prenatal uterine surgery scar.

Fetuses who underwent MMC correction were born at an average gestational age of 34.1 weeks, with 13% being born before 30 weeks. In contrast, those who underwent postnatal correction were born at an average of 37.3 weeks, with none born before 30 weeks. While there was no significant difference in the rates of neonatal adverse effects between the groups, one-fifth of the prenatal repair group experienced respiratory problems related to prematurity. Other complications of prematurity occurred at similar rates in both groups.

There were two perinatal deaths in each group, both on the fifth postoperative day. In the prenatal repair group, there was one fetal demise at 26 weeks and one neonatal death at 23 weeks due to extreme prematurity. In the postnatal repair group, there were also two deaths in infants who underwent CSF placement and were secondary to severe symptoms of Chiari II malformation. The shunt placement rate was significantly higher in the postnatal surgery group in comparison to the prenatal surgery group (82% vs. 40%, *p* < 0.001). Furthermore, the presence of hindbrain herniation at 12 months of age was lower in the fetal surgery group (4% vs. 36%) [21]. It was also noted at long-term follow-up at 30 months that the prenatal surgery group was more likely to walk without assistance (*p* = 0.01) with improvement of function in two or more anatomic levels of lesions [7,8,9].

Since the publication of the MOMS trial outcomes, numerous fetal MMC treatment centers have spread throughout the world. Open fetal MMC correction in the post-MOMS era has recently been reevaluated. Maternal and neonatal data were collected on 100 patients who underwent surgery over five years [21]. The average gestational age at evaluation was 21.9 weeks, and at repair, 23.4 weeks. The mean gestational age at delivery was 34.3 weeks, with 54.2% of patients delivering at or beyond 35 weeks. The perinatal loss rate was 6.1%, and 90.8% of women delivered at the same hospital where the surgery was performed. Additionally, 3.4% of women required transfusions. The most frequent complications were premature labor (37.5%), premature rupture of membranes (PROM) (32.3%), and chorioamniotic membrane separation (22.9%). Among the 80 neonates evaluated, 55% showed functional improvement by one or more levels compared to the prenatal anatomical assessment. Furthermore, 71.1% of neonates showed no evidence of posterior brain herniation on MRI, and only two required ventriculoperitoneal (VP) shunt placement before discharge. The authors conclude that in a single, experienced center, the maternal–fetal outcomes are comparable to the results from the MOMS trial [22].

Following the MOMS trial, fetal repair has become the standard surgical treatment for MMC, and numerous fetal treatment centers in the United States and around the world have presented case series with similar results [23,24,25,26,27,28]. In an attempt to reduce maternal morbidity, the refinement of the open repair technique with a mini-hysterotomy has reduced the risk of PROM and premature birth [29]. There has been increasing interest in the use of fetoscopic correction for MMC to further reduce the risks of hysterotomy. There remains a need to further standardize and refine the technical quality of fetal placode closure and expand the use of fetal surgery beyond specialized centers [30].

## 3. Fetoscopic MMC Repair

Open fetal repair has proven to be superior to traditional postnatal repair in reducing the need for VP shunt placement and improving motor outcomes at 30 months of life. However, it remains a highly invasive procedure, requiring both maternal laparotomy and hysterotomy [6]. As in many surgical fields, there has been a shift toward minimally invasive approaches in fetal surgery. Fetoscopic techniques for MMC repair were developed to minimize the maternal risks that were seen with open MMC repair.

The first fetoscopic MMC repair performed on a fetus was conducted in the United States at Vanderbilt University in the late 1990s [31]. Unfortunately, two of the four fetuses died from extreme prematurity and intraoperative placental abruption. Three more cases were performed at the University of California, San Francisco, with similar technical challenges and complications [32]. Only 1 out of 13 patients underwent complete fetoscopic MMC repair with a two-layer closure (2LC). Postnatal repair was required due to CSF leak as well as a ventriculoperitoneal (VP) shunt. These initial attempts led to hesitation to continue with the development of fetoscopic techniques in MMC repair. However, Kohl et al. in Germany demonstrated that fetal repair using percutaneous fetoscopy resulted in neonatal outcomes comparable to those seen in the MOMS trial and post-MOMS studies but failed to significantly reduce the rate of maternal complications [33,34,35,36].

A comparison of early reports from centers performing fetoscopic MMC repairs with those performing open repairs revealed that the fetoscopic technique had longer operative times and a steeper learning curve. This was associated with a significantly increased risk of PROM, oligohydramnios, and earlier gestational age at birth. Additionally, the fetoscopic approach often failed to achieve a reliable “water-tight” closure of the defect, leading to complications such as postnatal CSF leakage, the need for postnatal MMC revision, and increased rates of VP shunt placement [19,31,32,33,34,35,36,37,38,39].

Belfort et al. reported their single-institution experience with an exteriorized uterus two-port fetoscopic technique [40]. A 3LC technique was subsequently incorporated based on the noted benefits of multilayer closure [41]. Inclusion and exclusion criteria were established according to the MOMS trial. The uterus was exteriorized via a laparotomy, and two 12 Fr ports were used to attain access. The membranes were plicated to the uterine wall using 2-0 poly-4-hydroxybutyrate sutures to reduce the risk of chorioamniotic membrane separation. The placode was dissected free and allowed to drop into the spinal canal. Similar to in the MOMS trial, a patch was used to minimize the risk of cerebrospinal fluid (CSF) leakage and tethered cord, but instead of the Duragen^®^ patch, a bovine collagen patch (Durepair^®^) was applied. The dura and myofascial layers were sutured close in interrupted fashion. Lateral relaxing skin incisions were used if needed to approximate the skin in the midline with interrupted sutures in order to provide a “water-tight” closure. This technique yielded more favorable maternal and fetal outcomes compared to previous fetoscopic case series, including a lower rate of PROM, higher rates of vaginal delivery, and more neonates delivering closer to term. Additionally, the technique demonstrated a lower rate of CSF leakage and a higher rate of hindbrain herniation resolution at 6 weeks postoperatively [41]. However, the study had limitations, including a lack of long-term postnatal outcome data. Moreover, it did not show reduced operative time compared to other fetoscopic techniques, due to the learning curve associated with a new surgical skill.

Sanz-Cortes et al. reported the outcomes of the first 12 months of infants who underwent fetoscopic open repair of MMC in the international registry [42]. The International Fetoscopic Neural Tube Defect Repair Consortium was composed of 14 different centers from the United States, South America, Europe, and South Africa. During the prospective study, 300 patients met the eligibility criteria and underwent fetoscopic repair, with 285 successfully completing the procedure. The registry highlighted significant variations in technique and surgical team expertise across the different centers. Some centers had performed fewer than 5 cases, while others had completed more than 100 during the study period. Techniques varied, including the use of 2, 3, or 4 trocars depending on the center’s preference and whether a plication stitch was used to tack the membrane to the uterine wall. Closure methods also differed, ranging from single-layer closure with a patch to three-layer closure with or without a relaxing incision, and running versus interrupted sutures. This lack of standardization in technique among the 14 centers made it challenging to demonstrate that one fetoscopic approach was superior to the other in comparison to open fetal MMC repair. The duration of fetoscopic repair was 2.6 times longer in comparison to the open approach established in the MOMS trial. One notable difference was the mode of delivery: one-third of mothers in the fetoscopic registry delivered vaginally, compared to universal cesarean deliveries in the MOMS and post-MOMS trials (*p* < 0.01). In addition, there was no evidence of the thinning or dehiscence of the hysterotomy scar at the time of cesarean section delivery for the mothers in the fetoscopy registry in comparison to the MOMS and post-MOM trial (*p* < 0.01). However, no significant differences were observed in the neurological outcomes of infants between the fetoscopic registry and the MOMS trial [42].

As a follow-up to their prior study, Sanz-Cortes published more recent outcomes in the school-age years for neonates with MMC repaired at a single institution from December 2011 to July 2021 [43]. About half of the fetoscopic cases were delivered vaginally at a median gestational age of 38.1 weeks with no reported case of uterine rupture. Both were significantly better when compared to the cohort of mothers who underwent either hysterotomy or postnatal repair (*p* < 0.001). Long-term outcomes were comparable to those seen in neonates who underwent fetal hysterotomy repair. They had a significantly less need for the treatment of hydrocephalus and improved motor function at 1 year but a higher rate of spinal inclusion cyst surgery and symptomatic tethered spinal cord in comparison to the postnatal cohort. The higher incidence of tethered spinal cord was seen more commonly in neonates that had undergone single-layer fetal MMC repair, which led the investigators to abandon this technique for the three-layer closure.

## 4. Future Directions of MMC Repair

Further work has been performed to optimize the minimally invasive approach of fetal MMC repair, with particular interest in finding an alternative to the steep learning curve of fetoscopy. There are hopes that the advances in the field of robotics will achieve the finesse and precision required for fetal tissue manipulation, which can be challenging with traditional laparoscopic instruments [44]. Robotic surgery has the advantage of articulating hand motions and manipulating delicate tissues similarly to open procedures, albeit without the benefit of tactile feedback. The enhanced dexterity, motion stabilization, and three-dimensional optics have attracted many surgical specialties to integrate surgical robots into their procedures, especially in confined working spaces. These additional benefits have played an important role in improving surgeon comfort in learning these new skills when compared to laparoscopic techniques. However, despite these benefits, the application of robotic surgery in the fetus remains experimental, largely limited to animal studies, mainly due to the lack of appropriately sized instruments for in utero repair [45,46,47,48].

There have been recent advances in fetal tissue engineering research to create a tissue graft of stem cells to correct the anatomic defect of MMC [44,49,50,51,52,53,54,55]. This remains in the experimental phase due to the immunological barriers seen with stem cell transfers. One such initiative, the CuRe Trial (Cellular Therapy for In Utero Repair of Myelomeningocele) at the University of California, Davis, is currently recruiting mothers eligible for fetal surgery based on MOMS criteria. In this phase I clinical trial (NCT04652908), patients are randomized into cohorts, with the treatment group receiving a placental mesenchymal stem cell (PMSC-ECM) graft. The outcomes from this trial are still pending [49]. Meanwhile, at the University of Texas at Houston, the use of human umbilical cord as a skin patch for fetal MMC repair is being investigated. An animal study with an ovine model showed improved sensorimotor function in sheep repaired with the human umbilical cord patch [52,56]. This is presently being studied in a phase I clinical trial (Fetoscopic NEOX Cord 1K Spina Bifida Repair, NCT04243889).

## 5. Discussion

Open fetal MMC repair is currently the standard-of-care treatment for a selective group of patients, as seen in the MOMS trial. Its standardized approach requires a maternal laparotomy, hysterotomy, placode dissection and untethering, and multilayered water-tight closure [19]. The field of fetal surgery will advance with breakthroughs in minimally invasive technology that will refine fetoscopic techniques and improve outcomes for both mother and fetus. Thus far, there is a wide variety of technical approaches used by each center, as seen in the findings of the International Fetoscopic Neural Tube Repair Consortium [42]. Yet, fetoscopic repair is frequently offered as a safe alternative to open hysterotomy repair, when the same operation is not necessarily being performed on the fetus. The maternal benefits of the fetoscopic approach should not be at the expense of the fetus receiving effective repair.

Furthermore, the prolonged operative time of fetoscopic repair is an area of concern. Studies have reported operative durations ranging from 145 to 450 min for the transuterine fetoscopic approach [42] and 98 to 480 min for the percutaneous approach [33,57], compared to 54 to 130 min for open fetal repair [22,23]. This suggests not only extended time for carbon dioxide insufflation but also fetal exposure to general anesthesia. Animal studies have shown that anesthetic agents mainly acting via the GABA and NMDA pathways have neurotoxic effects on fetal brain development [58]. While not confirmed in humans, the long-term effect of prolonged anesthetic exposure in these midgestation human fetuses remains to be investigated.

For fetoscopic MMC repair to be considered a suitable alternative for MMC patients, it must be shown to be equal or superior to open fetal repair in maternal, neonatal, and long-term neurological outcomes. Comparisons to the 10-year-old results of the MOMS trial are no longer sufficient. There have been remarkable advances in open fetal repair since the MOMS trial—multilayer closure, mini hysterotomy, etc. Comparisons of fetoscopic MMC repair with more contemporary outcomes of open fetal repair would be more appropriate.

**Figure 1:**
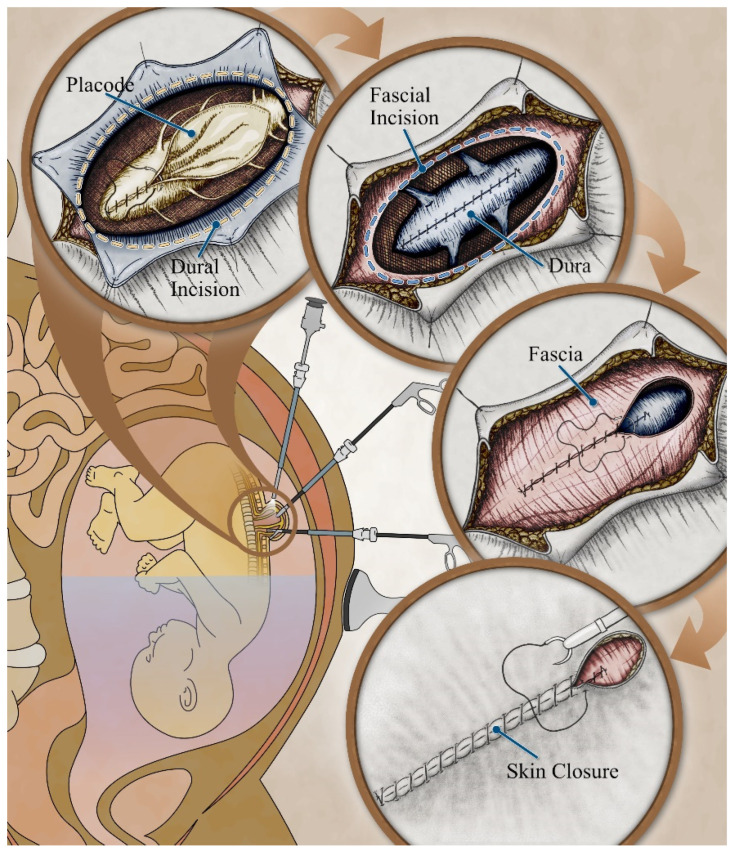
Diagram depicting fetoscopic hybrid multiport three-layer closure.

The MOMS trial took over 10 years to complete. The challenges of another randomized controlled trail to demonstrate equivalency or superiority of fetoscopic and open fetal repair of MMC are enormous. The prospective collection and analysis of registry data and outcomes may be the next best option. In order to generalize and truly compare the outcomes of fetoscopic MMC repair to open fetal MMC repair, the technique must be standardized across all fetal centers attempting the fetoscopic approach. Fetal surgeries should be performed at high-volume centers with a multidisciplinary team of specialists in pediatric neurosurgery, maternal–fetal medicine, anesthesiology, neonatology, and pediatric surgery. Surgical steps adapted from postnatal and open fetal repair should be employed in the fetoscopic approach (Figure 1).

Based on this, we recommend the technical steps described in Table 1, which our institution has formalized and implemented. The uterus would be exteriorized via maternal laparotomy, and the membranes, transfixed for access, as described by Belfort et al. [41,43]. A three-layer closure would be performed to achieve a water-tight closure. A dura patch would be utilized if the dura cannot be closed primarily or completely. A myofasical layer will be closed prior to skin closure. Attempts will be made to obtain a primary skin closure. Lateral relaxing skin incisions or rotational flaps can be used to achieve skin closure, and a skin patch can be used as a last resort. A three-port technique would provide the operating surgeon the flexibility and dexterity to gently manipulate the tissue and intracorporeal suture tying in an ergonomic fashion. This would help reduce the surgical time.

As advances continue to be made in the field and techniques modified, it is also important to have a standardized method of reporting surgical techniques utilized so any variations in treatment can be clearly identified (Table 2). This will enable adequate comparisons of different techniques to evaluate their impacts on maternal safety and fetal outcomes.

## 6. Conclusions

In summary, fetal surgery for MMC repair has made significant advances over the last 40 years with the development of novel techniques. However, for fetoscopic techniques to be considered comparable to open repair, the fetoscopic approach must not decrease maternal morbidity at the expense of an effective fetal closure. Merely reducing maternal morbidity without achieving the expected fetal benefit is a disservice to the fetus and unnecessary surgery for the mother. Three-layer closure has been shown to be effective in postnatal and open fetal MMC repair and should thus be the goal of fetoscopic repair. Any adjuncts or deviation should be considered experimental and disclosed to the patient as such and subjected to the rigors of clinical research. Such modifications should be reported in a standardized fashion for easy comparison. Furthermore, advances have been made in the approach and outcomes of open MMC repair since the MOMS trial such that comparing results with those of the MOMS trial (conducted more than a decade ago) is an exceptionally low bar. Fetoscopic surgery results should be compared to the more contemporary open MMC repair results.

## Figures and Tables

**Table 1:** Fetoscopic hybrid multiport three-layer closure technique: surgical steps for fetoscopic transuterine multiport three-layer closure.

Fetoscopic Hybrid Multiport Three-Layer Closure Technique
Exteriorization of the uterus via laparotomy and gentle manipulation of fetus into proper position.
2. Using ultrasound guidance, 2-0 absorbable monofilament suture is used to plicate the membrane to the uterine wall at each port site PRIOR to port insertion.
3. Via the Seldinger technique and ultrasound guidance, 10-12 Fr sheath ports are introduced for uterine access.
4. Amniotic fluid is removed, and carbon dioxide is insufflated via a heated humidifier.
5. Anesthetic cocktail is administered intramuscularly to the fetus.
6. Placode is dissected and dropped into the spinal canal.
7. Dura is mobilized laterally from the fascia and closed with a running 4-0 absorbable monofilament suture. A dural patch may be placed.
8. Skin is undermined, freeing the myofascial layer, which is then mobilized.
9. Myofascial layer is closed over the dura with a running absorbable monofilament suture
10. Skin is closed using a running 4-0 absorbable monofilament. A relaxing incision, 2 cm lateral from the defect, may be used to aid skin closure Rotational flaps can also be considered. A dermal graft may be used if primary skin closure cannot be accomplished.

**Table 2:** Proposed standard minimal reporting element for fetoscopic cases. The documentation of the standard elements of fetoscopic repair as well as any adjuncts or variation will be noted. The documentation of surgical techniques is recommended in order to standardize reporting from each institution.

Proposed Standard Minimal Reporting Elements for Fetoscopic Cases at Each Fetal Center
Percutaneous or laparotomy-assisted Exteriorization of the uterus: Yes or No Uterine membrane plication performed: Yes or No Number of fetoscopic ports used Use of humidified CO_2_: Yes or No Volume (mL) of CO_2_ infused Placode dissected and tubularized: Yes or No Dural closure performed or overlay patch was used. Any adjuncts to dural closure—stem cells, amnion patch, etc. Myofascial layer closure performed: Yes or No Method of skin closure: ☐ Primary suture closure ☐ Rotational flap ☐ Relaxing skin incision ☐ Patch

## Data Availability

No new data were created or analyzed in this study. Data sharing is not applicable to this article.
